# Hyperleptinemia Is Required for the Development of Leptin Resistance

**DOI:** 10.1371/journal.pone.0011376

**Published:** 2010-06-29

**Authors:** Zachary A. Knight, K. Schot Hannan, Matthew L. Greenberg, Jeffrey M. Friedman

**Affiliations:** Howard Hughes Medical Institute, Laboratory of Molecular Genetics, The Rockefeller University, New York, New York, United States of America; Louisiana State University, United States of America

## Abstract

Leptin regulates body weight by signaling to the brain the availability of energy stored as fat. This negative feedback loop becomes disrupted in most obese individuals, resulting in a state known as leptin resistance. The physiological causes of leptin resistance remain poorly understood. Here we test the hypothesis that hyperleptinemia is required for the development of leptin resistance in diet-induced obese mice. We show that mice whose plasma leptin has been clamped to lean levels develop obesity in response to a high-fat diet, and the magnitude of this obesity is indistinguishable from wild-type controls. Yet these obese animals with constant low levels of plasma leptin remain highly sensitive to exogenous leptin even after long-term exposure to a high fat diet. This shows that dietary fats alone are insufficient to block the response to leptin. The data also suggest that hyperleptinemia itself can contribute to leptin resistance by downregulating cellular response to leptin as has been shown for other hormones.

## Introduction

Body weight in mammals is controlled by a physiological system that balances energy intake and expenditure over the long term[1]. The core component of this system that signals the availability of body fat is the hormone leptin[2]. Leptin is secreted by adipocytes in proportion to their size and number, such that the concentration of leptin in the blood is proportional to the total amount of adipose tissue[3], [4]. Binding of leptin to its target neurons, which are expressed in the hypothalamus, brain stem and other brain regions, inhibits feeding and stimulates energy expenditure. Leptin thus functions as the afferent signal in a negative feedback loop that maintains a stable level of body fat reserves.

Leptin deficient mice (ob/ob) and humans are obese and hyperphagic[5], and, in these individuals, leptin replacement therapy induces dramatic weight loss[6], [7], [8], [9]. However most obesity is associated with elevated plasma leptin levels[3], [4], implying resistance to leptin's weight reducing effects[10]. The contribution of leptin resistance to obesity has also been established by the demonstration that hyperleptinemic animals and humans have a blunted response to exogenous leptin. Despite the importance of delineating the causes of leptin resistance, the cellular and molecular mechanisms responsible remain poorly understood.

The diet-induced obese mouse is a well characterized system for studying the development leptin resistance and pathogenesis of obesity. In this model, C57Bl/6J mice fed a high-fat diet (45% to 60% calories from fat) become progressively obese and hyperleptinemic over a span of 4 to 6 months. As these animals become obese, they lose the ability to reduce their food intake and body weight in response to leptin treatment. In the early stages of obesity, mice develop resistance to leptin delivered peripherally, but not centrally; this has been attributed to downregulation or saturation of the transport system that transports leptin across the blood-brain barrier[11], [12]. After long-term exposure to high-fat diet (>20 weeks), mice become resistant to leptin even when it is directly infused into the brain via the cerebral ventricle[10], [12], [13], [14]. In these animals, the first-order, leptin-responsive neurons have apparently lost the ability to activate the signaling pathways downstream of the leptin receptor.

How does exposure to a high-fat diet impair the leptin sensitivity of these neurons? Two models have been proposed. The first is that leptin resistance is caused by elevated plasma leptin levels, which result in chronic overstimulation of the leptin receptor and activation of negative feedback pathways that block further leptin signaling. This model is supported by the fact that leptin stimulates the expression of SOCS-3, a protein that directly inhibits leptin signaling[15], [16], [17], and that ablation of SOCS-3 in neurons enhances leptin sensitivity and protects against diet-induced obesity[18], [19], [20]. Moreover, targeted expression of a constitutively active form of STAT3, which is a key mediator of leptin signaling, is sufficient to induce leptin resistance in the hypothalamus[21]. This mechanism is analogous to the decrease in insulin receptor signaling that is associated with chronic insulin treatment and believed to result from the activation of negative feedback pathways such as serine phosphorylation of IRS-1[22].

An alternative explanation for the development of leptin resistance is that dietary fats themselves, rather than hyperleptinemia, are responsible. Fats could either directly block leptin signaling or activate cellular processes, such as endoplasmic reticulum (ER) stress and inflammation, that impair leptin responsive neurons[23], [24], [25], [26], [27], [28]. This model is supported by the fact that pharmacological or genetic modulation of fat metabolism in the hypothalamus has been shown to influence energy balance and leptin sensitivity[26], [29], [30]. Moreover, leptin resistance is known to develop most strongly in the arcuate nucleus of the hypothalamus, which relative to other regions of the brain has enhanced access to circulating nutrients[13]. In addition, it has been observed in some[10], [31], but not all[32], experimental settings that mice fed a high-fat diet fat do not consume more calories than mice fed a low-fat diet; this implies that dietary fat itself, rather than increased energy intake, may be responsible for leptin resistance in these animals.

In order to distinguish between these two possibilities, we separated the contributions of hyperleptinemia and dietary fat to the development of leptin resistance by (1) using osmotic infusion pumps to clamp the plasma leptin of ob/ob mice to the level found in lean wild-type animals over the long term, and then (2) measuring the leptin sensitivity of these animals after being placed on either a low- or high-fat diet.

## Results

**Figure 1:**
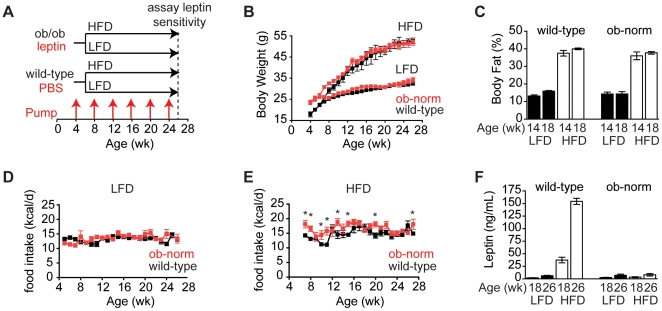
Energy balance in wild-type and ob-normalized animals on low and high-fat diets. **A**. Schematic of experimental strategy. Wild-type and ob/ob animals were implanted with pumps dispensing PBS or leptin, respectively, at 4 weeks of age, and pumps were replaced every four weeks as indicated by red arrows. Leptin sensitivity was assayed at 26 weeks of age. **B**. Body weight of wild-type (black) and ob-norm (red) mice on a high-fat or low-fat diet. **C**. Body fat percentage of wild-type and ob-norm mice at 14 and 18 weeks of age that were maintained on a high-fat diet (open bars) or low-fat diet (filled bars). **D**. Daily food intake for wild-type (black) and ob-norm (red) mice on a low-fat diet. p>0.05 for wild-type vs. ob-norm at all time points. **E**. Daily food intake for wild-type (black) and ob-norm (red) mice on a high-fat diet. * indicates p<0.05. **F**. Plasma leptin concentrations of wild-type and ob-norm mice at 18 and 26 weeks of age that were maintained on a high-fat diet (open bars) or low-fat diet (filled bars). All error bars are +/− SEM.

The development of central leptin resistance in C57Bl/6J mice requires exposure to a high-fat diet for 20 weeks[10], [14]. To establish the possible contribution of hyperleptinemia versus a high fat diet itself to the development of leptin resistance, we developed an experimental protocol in which, beginning at weaning, the plasma leptin levels of ob/ob mice could be fixed to the level of lean wild-type mice (∼5 ng/mL) for this duration (Figure 1a). We performed extensive dose response studies infusing leptin into ob/ob mice via osmotic infusion pumps and found that wild type plasma leptin levels of approximately 5 ng/mL in ob/ob mice could be achieved by delivering leptin at 150 ng/h. This infusion rate could also be stably maintained for longer than six months by replacement of the pumps every 28 days.

Pumps dispensing 150 ng/h leptin were implanted in male ob/ob mice at four weeks of age (hereafter referred to as “ob-norm”), and leptin treatment normalized the body weight of these animals within three weeks (Figure 1b). Identical pumps dispensing vehicle (PBS) were implanted in a control group of male wild-type mice. At six weeks of age, animals from each group were assigned to either a high-fat diet (60% of calories from fat) or a low-fat chow diet (13% of calories from fat) and maintained on this diet for 20 weeks with monthly pump replacements.

The body weight of the ob-norm and wild-type mice on the low-fat diet was similar throughout the course of the experiment (Figure 1b). There was no significant difference in average weekly food intake between the two groups (Figure 1d; 13.5±0.3 kcal/d for ob-norm versus 13.1±0.2 kcal/d for wild-type, p = 0.26) and their body fat percentages were similar when measured at 14 and 18 weeks (Figure 1c). Plasma leptin levels were measured periodically and, there was no significant difference in leptin levels between the two cohorts on the low-fat diet (Figure 1f; p>0.22). Interestingly, a small age-dependent increase in plasma leptin was observed in wild-type mice on a low-fat diet, and this was also observed in the ob-norm animals (2.6±0.4 ng/mL versus 1.9±0.4 at 18 weeks of age, and 7.0±2.3 versus 6.1±0.9 ng/mL at 26 weeks of age for ob-norm and wild-type, respectively; p<0.01 for the comparison between 18 and 26 weeks within either cohort). Because the only source of leptin in the ob-norm mice was pumps that delivered a constant dose, this small age-dependant increase likely reflects age-dependant changes in leptin metabolism or excretion.

Exposure to a high-fat diet resulted in obesity in both wild-type and ob-norm animals, and the time course of weight gain was indistinguishable in the two groups (Figure 1b). The body weights of wild-type and ob-norm animals were similar after 20 weeks on a high-fat diet (52.6±0.4 g in wild-type versus 52.1±1.8 g in ob-norm, p = 0.79). As expected, the plasma leptin levels of animals in these two groups were very different (Figure 1f). Wild-type animals became markedly hyperleptinemic as they became obese (37.4±5.6 ng/mL at 18 weeks of age and 154±6 ng/mL at 26 weeks of age), whereas the plasma leptin levels of ob-norm animals on a high-fat diet were the same as low-fat diet, chow fed controls (e.g., 7.0±2.3 ng/mL on a low-fat diet versus 8.1±2.3 ng/mL on a high-fat diet at 26 weeks of age, p = 0.75).

The nearly identical body weight trajectory of wild-type and ob-norm animals on a high-fat diet was somewhat unexpected: unlike wild-type animals, the weight gain of ob-norm mice is not restrained by increases in plasma leptin. We measured weekly food intake in both cohorts after exposure to a high-fat diet, and while the ob-norm animals did eat more food than wild-type animals at a subset of time points (Figure 1e; p<0.01), this slightly elevated food intake did not translate into increased body weight or adiposity (Figure 1c). These data show that wild-type and ob-norm animals display a very similar progression of diet-induced obesity in response to a high-fat diet, despite the fact that they maintain a ∼20-fold difference in plasma leptin levels.

**Figure 2:**
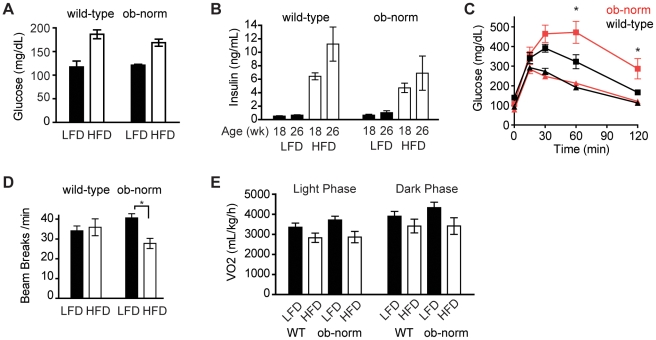
Glucose metabolism and energy expenditure. **A**. Plasma glucose concentrations in *ad libitum* fed wild-type and ob-norm mice that were maintained on a high-fat diet (open bars) or low-fat diet (filled bars). **B**. Plasma insulin concentrations of wild-type and ob-norm mice at 18 and 26 weeks of age that were maintained on a high-fat diet (open bars) or low-fat diet (filled bars). **C**. Plasma glucose concentrations in response to a glucose injection in wild-type (black) and ob-norm (red) mice. Triangles represent mice on a low-fat diet and squares represent mice on a high-fat diet. * indicates p<0.05. **D**. Average beam breaks per minute for wild-type and ob-norm mice maintained on a low-fat (filled bar) or high-fat (open bar) diet. **E**. Average oxygen consumption for wild-type and ob-norm mice in the light phase (open bars) and dark phase (closed bars).

Diet-induced obesity causes insulin resistance, whereas leptin improves glucose homeostasis independent of its effects on body weight[33], [34], [35], [36]. We therefore compared glucose metabolism in the four groups of animals in this experiment. Both wild-type and ob-norm animals on a high-fat diet developed hyperglycemia relative to low-fat diet controls (Figure 2a; p<0.001), and there was no significant difference in fasting blood glucose between cohorts on the same diet (p>0.15). We measured plasma insulin levels in all four groups at 18 and 26 weeks of age (Figure 2b). Both wild-type and ob-norm animals on a high-fat diet were hyperinsulinemic relative to low-fat diet controls (p<0.05), confirming that their hyperglycemia was a consequence of insulin resistance. By contrast, there was no significant difference in plasma insulin between cohorts on the same diet (p>0.24). These data indicate that the differences in blood glucose and insulin levels were determined by diet, not plasma leptin levels.

We performed glucose tolerance tests to measure the ability of each cohort to clear a bolus of sugar from the blood (Figure 2c). For mice on a low-fat diet, plasma glucose levels were indistinguishable between wild-type and ob-norm cohorts throughout the test (p>0.5). This confirms that leptin replacement at physiological levels was sufficient to normalize glucose homeostasis in the lean ob-norm animals. By contrast, both cohorts exposed to a high-fat diet showed delayed glucose clearance (Figure 2c; p<0.01). The magnitude of this impairment was greater in ob-norm animals relative to wild-type controls at 60 and 120 minutes (Figure 2c; p<0.01). Thus, while the high-fat fed wild-type and ob-norm animals have similar fasting hyperglycemia and hyperinsulinemia, the ob-norm animals have an additional defect in glucose metabolism that is revealed by glucose challenge suggesting that their relative leptin deficiency may further impair glucose metabolism of DIO mice.

Leptin-deficient ob/ob mice are hypoactive and have reduced energy expenditure. We therefore performed respirometry and activity measurements to compare the four groups of animals in this experiment (Figure 2). Activity was quantified by measuring beam breaks in three dimensions in animals that had been acclimated in metabolic cages. For the groups on a low-fat diet, there was no significant difference in average beam breaks between the wild-type and ob-norm groups (Figure 2d). For animals on a high-fat diet, there was a trend toward decreased activity in the ob-norm group, but this did not reach significance (35.9±4.3 breaks/min in wild-type versus 27.7±2.6 breaks/min in the ob/ob norm, p = 0.12). Similarly, we found that there was no significant difference in oxygen consumption, during either the light or dark phases, between wild-type and ob-norm cohorts on the same diet (Figure 2e).

Having established that wild-type and ob-norm animals have a similar body weight and physiology when maintained on the same diet, other than a modest impairment of glucose metabolism among ob-norm animals on a high fat diet, we next tested whether long-term exposure to a high- or low-fat diet affected the leptin sensitivity of these two groups. We used two assays for this purpose. First, in order to measure functional sensitivity to leptin, we tested whether a short-term leptin infusion could reduce food intake and body weight in each of the four cohorts. We did this by replacing the mini-osmotic pumps in all animals with pumps that delivered leptin at either the same rate (“vehicle”) or at a rate 450 ng/h higher. This results in a 4-fold increase in the leptin infusion rate in ob-norm animals (150 ng/h versus 600 ng/h leptin) and has previously been shown to result in a 4-fold increase in plasma leptin in lean wild-type animals[37]. We then measured food intake and body weight for 12 days, at which point the pumps were replaced with pumps dispensing leptin or PBS at the baseline rate.

**Figure 3:**
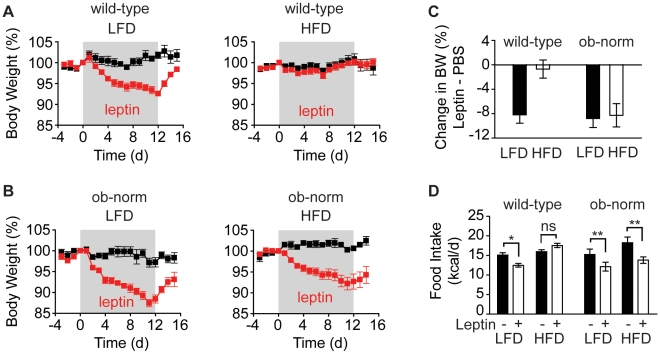
Leptin sensitivity in a 12-day infusion. **A**. Body weight of wild-type animals that received a leptin (red) or PBS (black) infusion for 12-days (gray block). **B**. Body weight of ob-norm animals that received a leptin (red) or PBS (black) infusion for 12-days (gray block). **C**. Difference in weight loss between leptin and vehicle for each cohort over the 12-day leptin infusion. Values are expressed as percentage change in body weight. **D**. Average daily food intake during the 12-day infusion for animals in each cohort.

As expected, wild-type mice maintained on a low-fat diet remained sensitive to exogenous leptin, reducing their food intake (15.1±0.6 kcal/d for vehicle versus 12.5±0.5 kcal/d for leptin, p<0.05) and showed a progressive reduction of body weight throughout the 12-day infusion (Figure 3). By contrast, wild-type mice that had been maintained on a high-fat diet for 20 weeks showed no reduction in food intake or body weight in response to the 450 ng/h leptin infusion (Figure 3). These results are consistent with previous reports showing that prolonged diet-induced obesity induces robust leptin resistance in mice[10].

ob-norm mice that had been maintained on a low-fat diet were sensitive to leptin, and showed a similar reduction of food intake as their wild-type counterparts, (15.2±1.4 kcal/d for vehicle versus 12.1±1.1 kcal/d for leptin, p = 0.08) and losing approximately 10 percent of their body mass over the course of the 12-day infusion (−2.8±1.1% for control versus −11.6±1.0% for leptin, p<0.05).

In contrast, and unlike wild-type controls on a high fat diet, ob-norm mice that had been exposed to a high-fat diet remained highly sensitive to exogenous leptin. These animals showed a significantly reduced food intake (18.3±1.5 kcal/d for vehicle versus 13.8±0.8 kcal/d for leptin, p<0.05) and body weight (0.6±0.6% for vehicle versus −7.3±1.8% for leptin, p<0.05) in response to the leptin infusion. Because wild-type and ob-norm animals differed only in their plasma leptin levels, this result confirms that hyperleptinemia is functionally required for the development of leptin resistance after long-term exposure to a high-fat diet.

**Figure 4:**
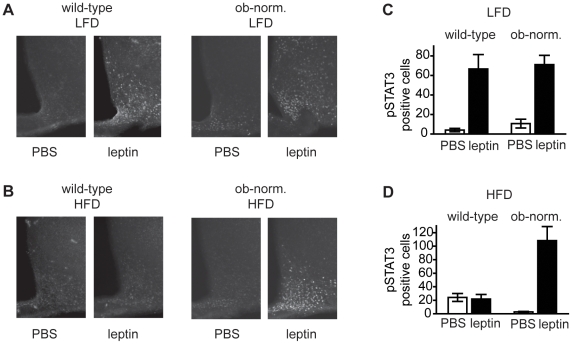
STAT3 phosphorylation in response to acute leptin injection. **A**. Staining for pSTAT3 in the mediobasal hypothalamus of mice on a low-fat diet given an intraperitoneal injection of either leptin or vehicle. **B**. Staining for pSTAT3 in the mediobasal hypothalamus of mice on a high-fat diet given an intraperitoneal injection of either leptin or vehicle. **C**. Quantitation of pSTAT3 positive cells in the arcuate nucleus for mice on a low-fat diet. **D**. Quantitation of pSTAT3 positive cells in the arcuate nucleus for mice on a high-fat diet.

In a second assay of leptin sensitivity, we next tested whether leptin could stimulate acute phosphorylation of STAT3 in neurons of the mediobasal hypothalamus among the four groups of mice[10], [13], [38]. Mice were given an intraperitoneal bolus of either vehicle (PBS) or leptin (2 mg/kg) and sacrificed 30 minutes later by cardiac perfusion. This dose of leptin has previously been shown to increase plasma leptin levels by 40-fold[10]. Brains were dissected, and STAT3 phosphorylation was quantified by immunohistochemistry (Figure 4). Wild-type animals maintained on a low-fat diet showed a robust increase in the number of pSTAT3 positive cells in response to leptin (Figure 4a; 4.0±1.7 cells for vehicle versus 67±15 cells for leptin, p<0.02). By contrast, wild-type mice maintained on a high-fat diet showed no increase in pSTAT3 positive cells in response to leptin (24.2±5.8 cells for vehicle versus 21.8±6.8 cells for leptin, p = 0.8), confirming that diet-induced obesity blunts the leptin-responsiveness of these first-order neurons. While wild-type mice on a high-fat diet were insensitive to exogenous leptin, these mice did have a modestly increased number of pSTAT3 positive cells at baseline compared to low-fat fed animals (Figure 4c, p<0.05). This supports prior suggestions that diet-induced obesity results in chronically elevated leptin signaling that cannot be further modulated by additional leptin[39], [40], [41].

We next tested ob-norm mice in the same assay. Ob-norm mice maintained on a low-fat diet showed increased STAT3 phosphorylation in response to leptin, and the magnitude of this increase was similar to wild-type controls (10.7±4.5 cells for vehicle versus 71±1.7 cells for leptin, p<0.01). Unlike wild-type animals on a high fat diet, ob-norm mice that had been maintained on a high-fat diet also showed a robust increase in STAT3 phosphorylation in response to leptin (2.7±0.8 cells for vehicle versus 108±21 cells for leptin, p<0.01). There was no indication that the leptin responsiveness of these high-fat diet fed, ob-norm mice was impaired in this assay relative to lean controls. These biochemical data are consistent with the physiological data from leptin infusion experiments and indicate that even long-term exposure to a high-fat diet is not sufficient to block the leptin-induced activation of JAK/STAT signaling in normoleptic animals. Instead, we conclude that hyperleptinemia is required for the development of leptin resistance in response to diet-induced obesity.

## Discussion

The mechanisms responsible for the development of leptin resistance have been the focus of many studies. As leptin resistance results in obesity and other metabolic diseases, agents which can re-sensitize the obese to leptin would have great therapeutic value. However, the molecular events responsible for the development of leptin resistance remain poorly understood, in part because of the numerous physiological and nutritional differences between lean and diet-induced obese animals. In principle, any of these differences could contribute to impaired leptin sensitivity.

In this study we asked whether one physiological parameter – elevated plasma leptin levels – is required for the development of leptin resistance in response to a high-fat diet. We focused on hyperleptinemia because negative feedback in response to excess signaling is a classical mechanism of hormone resistance (including insulin), and because there was already evidence supporting at least one molecular mechanism for negative feedback regulation of leptin (SOCS-3). These experiments were made possible by our having developed a protocol that could normalize the plasma leptin levels of ob/ob mice over the long-term using subcutaneous pumps. We found, consistent with previous studies, that leptin replacement in ob/ob animals is sufficient to normalize their body weight and other physiologic abnormalities, and furthermore that this normalization can be maintained for 6 months by replacing the pumps monthly. The data clearly show that the hyperleptinemia in mice fed a high fat diet is required for the development of leptin resistance. These results do not support the claim that a high-fat diet can block the response to leptin in ob/ob animals[42]. Recently, the peptide amylin has been shown to sensitize animals and humans to the effects of leptin[43], [44]. An important challenge will be to further understand the cellular basis of leptin resistance and the mechanism by which amylin can improve it.

In addition to its role in regulating energy balance in adult animals, leptin has trophic effects on hypothalamic neurons during early post-natal development[45]. We have shown that leptin treatment beginning at 4 weeks of age normalizes the food intake and adiposity of ob/ob mice on a low fat diet, but it remains possible that neonatal leptin deficiency could selectively influence the development of leptin resistance in response to a high-fat diet. Previous studies have found that leptin treatment of ob/ob mice rapidly normalizes the number of excitatory and inhibitory synaptic inputs to arcuate POMC and NPY neurons[46], suggesting that there is significant plasticity in the adult hypothalamus. Consistent with this, we have found that the leptin sensitivity of ob/ob mice subjected to long-term leptin replacement is indistinguishable from lean wild-type controls (in contrast to leptin-naïve ob/ob animals, which are hypersensitive to leptin). Nonetheless, certain hypothalamic projections are impaired in ob/ob mice, and leptin treatment of adult ob/ob animals does not correct these defects[45]. The significance of these anatomical differences to energy balance and leptin sensitivity remains unclear.

Recent work on leptin resistance has focused on the role of inflammation in the hypothalamus[47], [48]. This is based on the observation that fats, either when supplied in excess in the diet or administered centrally, can induce hypothalamic inflammation, and that genetic or pharmacological blockade of inflammatory signaling in the brain can improve leptin sensitivity[26], [27], [28], [49]. In addition, ER stress is a common byproduct of inflammation, and ER stress has been shown to contribute independently to the development of leptin resistance[23], [24], [25]. Our data are not inconsistent with a role for inflammation and ER stress in the development of leptin resistance. However, our data do imply that hyperleptinemia is required for any inflammation-mediated mechanism that blocks leptin signaling after exposure to a high-fat diet. In this respect, the fact that upregulation of SOCS-3 is one of the primary mechanisms by which inflammation has been proposed to inhibit leptin signaling suggests that hyperleptinemia and inflammatory cytokines may contribute to leptin resistance by activating a common set of pathways.

One unexpected finding from our study was that ob-norm animals, which had a fixed level of plasma leptin, nonetheless gained weight in response to a high-fat diet at a rate that was indistinguishable from wild-type animals on the same diet, and the two groups reached an identical body weight plateau (Figure 1b). If incremental changes in leptin significantly restrained body weight in response to excess dietary fat, then the ob-norm animals would be predicted to gain weight more quickly than wild-type controls and plateau at a higher body weight, if at all. The fact that this was not observed indicates that a leptin-independent mechanism specifies the body weight set point in animals fed a high-fat diet. While the nature of this mechanism is unclear, this observation is consistent with previous reports showing that transgenic mice that overexpress leptin in the liver (and therefore do not experience substantial increases in plasma leptin in response to obesity) nonetheless gain weight at a rate that is indistinguishable from wild-type animals when exposed to a high-fat diet[50]. Likewise, leptin infusion fails to prevent the weight gain that occurs when wild-type animals are exposed to a high-fat diet[51]. However, viral overexpression of leptin in the rat hypothalamus has been reported to potentiate the weight gain caused by a high-fat diet[52]. As body weight is regulated by numerous short and long-term signals, it will be important to establish how energy balance can be maintained in a leptin independent manner.

In summary, our finding that hyperleptinemia, and therefore excess leptin signaling, is required for the development of leptin resistance reinforces the view that understanding the changes in signal transduction within the sparse neuronal cell types that control energy balance will be essential to unraveling the mechanism of leptin resistance. One of the major challenges in studying leptin resistance has been the difficulty of accessing leptin's key target cells, which are a small subpopulation of neurons located primarily in the hypothalamus and brain stem. The development of BacTrap technology for neuron-specific profiling represents an exciting opportunity to begin to characterize the molecular changes that develop in these cells with acute and chronic hyperleptinemia, and these experiments are underway[53].

## Methods

### Ethics Statement

All procedures were carried out in accordance with the National Institutes of Health Guidelines on the Care and Use of Animals and approved by the Rockefeller University Institutional Animal Care and Use Committee (Protocol #09012).

### Animals: Diet and leptin normalization

Wild-type and ob/ob C57BL/6J mice were obtained from Jackson laboratories (Bar Harbor, ME). At 4 weeks of age, a micro-osmotic pump (model 1004; Durect, Cupertino, CA) dispensing either vehicle (PBS) for wild-type animals or leptin (150 ng/h in PBS) for ob/ob animals was implanted subcutaneously and mice were individually housed. Recombinant murine leptin was obtained from Amylin Pharmaceuticals (San Diego, CA). At 6 weeks of age, mice from each cohort were assigned to either remain on a low-fat chow diet (Picolab Rodent Diet 20, LabDiet, St. Louis, MO) or switched to a high-fat diet (D12492, Research Diets, New Brunswick, NJ). The low-fat diet contained 3.41 kcal/g (24.6% calories from protein, 13.2% calories from fat, and 62.1% calories from carbohydrates) and the high-fat diet contained 5.24 kcal/g (20% calories from protein, 60% calories from fat, and 20% calories from carbohydrates). Food intake and body weight were monitored weekly. Micro-osmotic pumps were replaced at week 8 and every 4 weeks thereafter to ensure continuous delivery of leptin or vehicle; model 2004 pumps were used after week 8. Body composition was measured by dual-energy x-ray absorptiometry (DEXA) densitometry (Lunar PIXImus 2, GE Medical Systems, Wisconsin).

### Glucose, Insulin, and Leptin Measurements

Glucose tolerance tests were performed on mice that had been fasted for 14 hr beginning at the onset of the dark cycle. The following day mice were given an intraperitoneal injection of an aqueous solution of 20% glucose (6.25 µL/g body weight) and blood glucose was measured from the tail vein at 0, 15, 30, 60, and 120 min using an Ascensia Elite XL glucometer (Bayer HealthCare, Tarrytown, NY).

Plasma hormone levels were measured using ELISA kits for leptin (R&D Systems, Minneapolis, MN) or insulin (Mercodia, Winston Salem, NC) from blood collected from the tail vein of *ad libitum* fed animals.

### Respirometry and Activity Measurements

Mice were individually housed in Oxymax metabolic cages (Columbus Instruments, Columbus, OH) with *ad libitum* access to food and water. Gas consumption and movement was recorded for 3–4 days. Animals that stopped eating or drinking during this interval or that lost significant body weight (>2 g) were excluded from the analysis.

### Leptin Infusion Assays

To measure the sensitivity of animals to short-term leptin infusion, the micro-osmotic pump in each animal was replaced with a 14-day pump (Model 2002, Durect) dispensing leptin at a rate 450 ng/h above baseline. This means that for wild-type animals, pumps delivering PBS were replaced with pumps delivering leptin at 450 ng/h, and for ob/ob animals, pumps delivering leptin at 150 ng/h were replaced with pumps delivering leptin at 600 ng/h. Body weight was recorded daily and food intake every 6 days. After 12 days pumps were removed and replaced with pumps dispensing leptin at baseline (PBS for wild-type animals and 150 ng/h for ob/ob animals).

### Immunohistochemistry

Mice were injected with either leptin (2 mg/kg, intraperitoneal) or vehicle (PBS). 30 min after injection animals were anesthetized with isoflurane and perfused with 10% neutral buffered formalin (Sigma, St. Louis, MO). Brains were removed by dissection and soaked in formalin overnight at 4°C. 50 µm sections were prepared and stained for pSTAT3 as follows[10]. Free-floating sections were treated with 1% H_2_O_2_+1% NaOH in water for 10 min, followed by 0.3% glycine in PBS for 10 min, and 0.03% SDS in PBS for 10 min. Sections were then exposed to blocking solution (PBS containing 0.1% Triton X-100/2% Goat Serum/3% BSA) for 1 hr. Phospho-STAT3 (Tyr 705) antibody (#9131, Cell Signaling, Danvers, MA) was diluted 1:1000 into blocking solution and sections were stained for 48 hr at 4°C. Sections were then washed (3 times, 20 min) and incubated with secondary antibody for 2 hr (Alexa 488 conjugated goat anti-rabbit antibody; Invitrogen, Carlsbad, CA). Sections were washed (3 times, 20 min), mounted on microscope slides, and photographed.

To quantitate the number of stained cells, a 300×300 pixel section was removed from the region representing the arcuate nucleus in each image. The number of positive cells was then counted by an observer blinded to the sample identity.
